# Contrasting Metabolism in Perenniating Structures of Upland and Lowland Switchgrass Plants Late in the Growing Season

**DOI:** 10.1371/journal.pone.0105138

**Published:** 2014-08-18

**Authors:** Nathan A. Palmer, Aaron J. Saathoff, Christian M. Tobias, Paul Twigg, Yuannan Xia, Kenneth P. Vogel, Soundararajan Madhavan, Scott E. Sattler, Gautam Sarath

**Affiliations:** 1 United States Department of Agriculture-Agricultural Research Service, Grain, Forage and Bioenergy Research Unit and Department of Agronomy and Horticulture, University of Nebraska, Lincoln, Nebraska, United States of America; 2 United States Department of Agriculture-Agricultural Research Service, Genomics and Gene Discovery Research Unit, Western Regional Research Center, Albany, California, United States of America; 3 Biology Department, University of Nebraska, Kearney, Nebraska, United States of America; 4 Center for Biotechnology, University of Nebraska, Lincoln, Nebraska, United States of America; 5 United States Department of Agriculture-Agricultural Research Service, Retired, Lincoln, Nebraska, United States of America; 6 Department of Biochemistry, University of Nebraska, Lincoln, Nebraska, United States of America; Universidade de Sao Paulo, Brazil

## Abstract

**Background:**

Switchgrass (*Panicum virgatum* L.) is being developed as a bioenergy crop for many temperate regions of the world. One way to increase biomass yields is to move southern adapted lowland cultivars to more northern latitudes. However, many southerly adapted switchgrass germplasm can suffer significant winter kill in northerly climes.

**Materials and Methods:**

Here, we have applied next-generation sequencing in combination with biochemical analyses to query the metabolism of crowns and rhizomes obtained from two contrasting switchgrass cultivars. Crowns and rhizomes from field-grown lowland (cv Kanlow) and upland (cv Summer) switchgrass cultivars were collected from three randomly selected post-flowering plants. Summer plants were senescing, whereas Kanlow plants were not at this harvest date.

**Results:**

Principal component analysis (PCA) differentiated between both the Summer and Kanlow transcriptomes and metabolomes. Significant differences in transcript abundances were detected for 8,050 genes, including transcription factors such as WRKYs and those associated with phenylpropanoid biosynthesis. Gene-set enrichment analyses showed that a number of pathways were differentially up-regulated in the two populations. For both populations, protein levels and enzyme activities agreed well with transcript abundances for genes involved in the phenylpropanoid pathway that were up-regulated in Kanlow crowns and rhizomes. The combination of these datasets suggests that dormancy-related mechanisms had been triggered in the crowns and rhizomes of the Summer plants, whereas the crowns and rhizomes of Kanlow plants had yet to enter dormancy.

**Conclusions:**

Delayed establishment of dormancy at more northerly latitudes could be one factor that reduces winter-survival in the high-yielding Kanlow plants. Understanding the cellular signatures that accompany the transition to dormancy can be used in the future to select plants with improved winter hardiness.

## Introduction

Switchgrass (*Panicum virgatum* L.), a perennial C_4_-grass native to the continental USA, is being developed as a major biomass feedstock for use in temperate regions [1]. Latitudinal adaptation has resulted in ecotypes with distinguishable genetic make-up [2] and differential responses to photoperiod [3]. Moving switchgrass germplasm more than one hardiness zone north or south from its native adaptation zone or latitude generally results in significantly reduced biomass yields. This is caused by early flowering in the northern adapted plants when moved to southern latitudes and reduced winter survival of southern plants when grown in northern latitudes because of delayed flowering and senescence [3].

For switchgrass, perenniality and sustainability appear to be strongly interlinked and are significantly influenced by photoperiod [3], [4]. Photoperiodic cues likely influence the orderly developmental transition from spring emergence of shoots through the senescence of the aerial parts of the plant and the imposition of dormancy in the below-ground parts of the plant post seed ripening [5]. As with other perennial warm-season grasses, the above ground parts of the switchgrass plant senesce at the end of the growing season, whereas the below-ground tissues, (comprised of the crowns, rhizomes and roots), transition to a dormant state. Tiller buds originating from the crowns and rhizomes are the source of the eventual regrowth of the above ground portions of the plant in the following growing season. Little is currently known at a molecular or cellular level on these aspects of switchgrass biology; however, if these processes can be understood it will be possible to improve both breeding and management of this crop. This knowledge can be used not only to improve the sustainability of switchgrass and other temperate perennial biomass grasses, but also potential perennial grain crops [6] which will face similar trade-offs of maximizing grain yield while maintaining crown and rhizome vitality. Seasonal senescence and dormancy has been studied in a number of other species [7]–[10], and these studies offer a scaffold to interpret data obtained from understudied species such as switchgrass.

This study focused on two tetraploid cultivars, namely, “Summer” that is adapted to the northern Great Plains of the USA and a contrasting cultivar “Kanlow” that is adapted to the southern latitudes of the USA. The cultivar Summer was developed at Brookings, South Dakota (∼44°N), USA from a native collection that originated from southeast Nebraska, USA and was first released in 1963 [11]. The cultivar Kanlow was also first released in 1963 from Manhattan, Kansas (∼39°N), USA and was developed from 200 plants originally sourced from a lowland site near Wetumka, Oklahoma, USA. Kanlow plants display adaptation to wet sites [11]. There is about a 26 cm differential in average rainfall between SE Nebraska (origin of cv Summer, drier) and Wetumka, OK (origin of Kanlow, wetter). Although we cannot rule out environmental-specific transcriptional differences between the two populations, our intention was to focus on understanding any stage specific differences. Little, if any, selection pressure was applied during the generation of the cultivars, and as with most switchgrass cultivars, both Summer and Kanlow are synthetic populations that display diversity for many plant characteristics including height, leaf morphology, phenology and genotype as has been described in other publications [1]–[3], [5], [12]. Tetraploid switchgrasses exhibit higher yields than octaploid switchgrasses, and are providing germplasm for the development of improved plant materials for the biofuel sector [13], [14]. Within the tetraploid switchgrasses, the lowland cultivars such as Kanlow out-yield upland tetraploids such as Summer, but suffer from significant winter-kill in more northerly latitudes limiting their potential to be grown in these regions [1], [3]. The aims of this study were to utilize RNA-seq with some biochemical analyses to develop knowledge into the metabolism of crown and rhizome tissues near the end of the growing season. The combination of these unbiased datasets obtained from the crowns and rhizomes from field-grown plants suggests that dormancy-related mechanisms had been triggered in the crowns and rhizomes of the Summer plants, whereas the crowns and rhizomes levels had yet to enter dormancy. Recently, RNA-seq has been used to map transcripts and discover single nucleotide polymrophisms (SNPs) in several different populations of switchgrass [15]. In this study the authors were able to efficiently map a large number of sequencing reads and identify over one million SNPs. A similar approach has been used in our study to understand the transcriptomes in the two contrasting switchgrass cultivars.

## Materials and Methods

### Plant Materials, Growth Conditions and Selection of Harvest Date

Stands of switchgrass cv Summer and cv Kanlow were established in the fields of the University of Nebraska-Agricultural Research Division near Mead, NE (∼41°N) in 2009, using seedlings raised in the greenhouse and transplanted to the fields. Each plot (1 m×1.2 m) contained 12 closely-spaced plants to mimic sward densities. Plots were in a field that measured approximately 35 m×30 m [16]. Agronomic management was as described earlier [17]. Plants were not irrigated and depended on ambient rainfall and stored soil moisture for growth. Climatic conditions for a few weeks prior to, and after collection are shown in Figure S1 in File S1. Daytime temperatures were generally greater than 20°C for the ∼4 weeks prior to harvest and low temperatures were generally 10° to 15°C. Three continuous days of rainfall were recorded three days prior to harvest. Soils were moist at harvest (Figure S1 in File S1).

At this location, Summer plants reach 50% anthesis generally by early July and reach physiological maturity by early-mid September. In contrast, for Kanlow plants, 50% anthesis is reached by mid-August and physiological maturity in late September/October. We did not control for plant size (although Kanlow plants were always larger than Summer plants), or measure above-ground yields. Individual plants planted in 2009 were still distinct in 2010, and the crowns and rhizomes were collected from three individual plants of each cultivar as described earlier [16] on September 29, 2010. Data were not collected on the above ground material except to visually verify seed developmental stage and if there were obvious signs of plant senescence (leaf yellowing, tiller death, etc.). Such signs of senescence were present in Summer plants, but were not observed for the Kanlow plants. This developmental state was selected as a starting point to understand the changes in the below-ground metabolism of switchgrass that impacted transition into winter dormancy. At harvest, tissues were flash frozen in liquid nitrogen and later pulverized cryogenically in order to obtain high yields of total RNA [16].

### RNA-Seq

Total RNA was extracted from tissues with TRIzol Reagent (Invitrogen Corp, Carlsbad, CA), followed by further purification by Qiagen RNeasy Mini Kit (Qiagen, Valencia, CA). Sequencing libraries were prepared from poly(A)+RNA using the Illumina mRNA-Seq Sample Prep Kit according to the manufacturer's instructions. Libraries were then sequenced on the Illumina Genome Analyzer IIx using two 36-cycle sequencing kits to read 75 nucleotides of sequence from a single end of each insert, by the v8 protocol (Illumina Inc, San Diego, CA). Each sample was analyzed on a single lane. The purity, integrity, and profile of extracted total RNA was verified on an Agilent 2100 BioAnalyzer (Agilent Technologies, Inc., Santa Clara, USA), and for concentration using a NanoDrop instrument (Thermo Scientific, Waltham, USA). Once generated, quality of the libraries was verified by gel electrophoresis and on a NanoDrop spectrophotometer. The PhiX virus DNA library was used as a control in a sequencing lane on each flowcell run to monitor sequencing operation. Obtained sequences were trimmed for adapter sequences and verified for quality using the software available with the instrument.

### Mapping and Differential gene expression analysis

Illumina reads were mapped to the draft switchgrass genome available at www.phytozome.net [18], using splice-junction aware Tophat2 (version 2.0.11) [19], with Bowtie2 (version 2.2.3) [20] alignment. Default parameters were used and reads flagged as having multiple alignments were not included in gene expression. Gene expression counts were calculated using featureCounts [21], a script included as part of the Subread (version 1.4.4) [22] analysis package, and the gene annotation file provided with the draft genome release. Differential expression analysis was conducted using the program edgeR (version 3.4.2) [23] in R [24]. Very low expression genes were filtered out of the data set prior to analysis by requiring 1 count per million (cpm) in at least three of the six samples. An FDR threshold of 0.05 was used to determine differentially expressed genes. Variance stabilizing transformation values, calculated using the DESeq2 (version 1.2.8) [25] package in R, were used for gene expression quantities in further downstream analyses.

### Genome Functional Annotation

Draft genome nucleotide transcript sequences were functionally annotated by sequence similarity using the program Blast2GO [26] similarly to our earlier work [16]. Blast2GO annotations with an e-value threshold of 1×10^−15^ or lower resulting from a Blastx search against the NCBI non-redundant protein database were used in all the subsequent analyses, including Enzyme Commission (EC) (http://www.chem.qmul.ac.uk/iubmb/enzyme/) and KEGG pathways [27], [28].

### Gene Set Enrichment Analysis

Gene Set Enrichment Analysis was done using GSEA from the Broad Institute [29]. Gene sets were created from Blast2GO generated KEGG pathway assignments.

### Metabolite Analyses

Aliquots (100±2 mg) of ground crown and rhizome tissue were extracted for metabolites, generally following a previously described protocol [30]. Metabolites were separated and identified (where possible) by GCMS [31] using the Agilent Fiehn GC/MS Metabolomics RTL library as the basis for instrument operation and metabolite identification. Metabolite identification was accomplished using the Fiehn libraries in Chemstation and in the supplied version of AMDIS. Metabolites were extracted from 100 mg of tissues with 350 µL 100% methanol and 20 µL of 0.2 mg mL^−1^ ribitol in water, as an internal standard. Samples were then heated in a 70°C heat block for 15 minutes. After heating, 1 volume (370 µL) DI water (18 MΩ) was added and each tube was vigorously mixed and then centrifuged at 14,000 RPM for 10 min. The supernatants were placed into new 1.5 mL tubes, and polar and nonpolar metabolites separated by two chloroform washes (300 µL each time). The final upper phase (methanol and water) was carefully pipetted into a new microfuge tube and 25 µL aliquots from each tube were then placed into 2 mL glass vials. Next, 5 µL of 0.4 mg mL^−1^ myristic-d_27_ acid (#366889 Sigma-Aldrich Co.) in 100% methanol and 20 µL of 0.2 mg mL^−1^ docosane (#134457 Sigma-Aldrich Co.) in dichloromethane were added to each vial; these were used as a retention locking compound and internal standard, respectively. The vials were evaporated to dryness under vacuum (Labconco Inc.). The dried samples were derivatized using 30 µL of 40 mg mL^−1^ methoxyamine hydrochloride at 37°C for three hours followed by 30 min. of trimethylsilylation at 37°C using 90 µL *N*-methyl-*N*-(trimethylsilyl)trifluoroacetamide (Thermo Scientific Inc.). The derivatized sample was placed into a new 2 mL glass vial with an insert due to the small volumes. The GC column was a 30-meter, 0.250 mm I.D. HP-5MS column (Agilent Technologies, Inc.). The mass selective detector was auto-tuned regularly with perfluorotributylamine as recommended by the manufacturer and operated over a *m*/Q scan range of 50–600. Temperature settings were 250°C for the injector and MS source and 290°C for the transfer line. The initial oven temperature was 60°C, which was increased at a rate of 10°C per minute to 325°C. Helium was the carrier gas and the flow rate was 1.1 mL min^−1^. The injector was operated in splitless mode. Since only major metabolites were of interest, features that had a mean major ion count of less than 1.0×10^5^ across both genotypes were discarded. Also, features that were missing in more than 10 of the 27 GC-MS runs per cultivar were flagged; features that were flagged this way across both cultivars were eliminated from the data set since they were not reliably detected across the biological and technical replicates. After this filtering, a total of 418 features remained in the data set, although not all of these features could be reliably identified by Chemstation or AMDIS.

### Enzyme, Protein and Immunoassays

Total soluble protein was extracted by sonicating approximately 100±2 mg of pulverized crown and rhizome tissue with 600 µL of Tris-HCl buffer, pH 8.0 containing a plant protease cocktail (Sigma P9599) with 5 mM DTT for cinnamyl alcohol dehydrogenase (CAD) or with 300 µL of Tris-HCl buffer, pH 8.0, without DTT for ascorbate peroxidase (AscPx) and caffeoyl-*O*-methyl transferase (COMT). For phenylalanine ammonia lyase (PAL), tissues were extracted using 0.1 M borate buffer, pH 8.8. Crude homogenate was desalted using a spin column (Thermo Scientific 89889, 2 mL columns) and used directly for enzyme assays. Protein content was measured using a dye-binding assay (Pierce 660 nm Protein assay kit) using BSA as a standard. Gel electrophoresis, protein transfer and immunodetection were as described previously [32]. Chemiluminescence was detected using a BioRAD Chemidoc system. Enzyme activities were analyzed as described previously [32]–[34].

### Statistical Analyses

Statistical analysis of metabolite data was conducted using SAS 9.3 (SAS Institute, Inc., Cary, NC) and JMP 9.0 (SAS Institute Inc., Cary, NC). Pairwise comparisons across the two cultivars used the Wilcoxon rank-sum test in PROC NPAR1WAY, and the raw p-values were adjusted in PROC MULTTEST to correct for multiple comparisons using the false discovery rate [35]. JMP 9.0 was used to conduct principal component analysis and generate the color map. ANOVA testing was performed on other datasets using statistical routines available in EXCEL.

## Results

### 80% of all reads map to the switchgrass draft genome

The mapping summary for the three biological replicates obtained from field grown Summer and Kanlow plants are shown in Table S1 in File S1. Each sample yielded approximately 24–27 million reads, of which greater than 77–83% were mapped to the 0.0 early-release version of the switchgrass genome. Functional annotation of the PviDraft0 genome by Blast2GO resulted in the annotation of 45,487 gene models at an e-value threshold of 1×10^−15^ or lower (Table S2 in File S1).

### Transcriptomes of Summer and Kanlow crowns and rhizomes are significantly different

**Figure 1:**
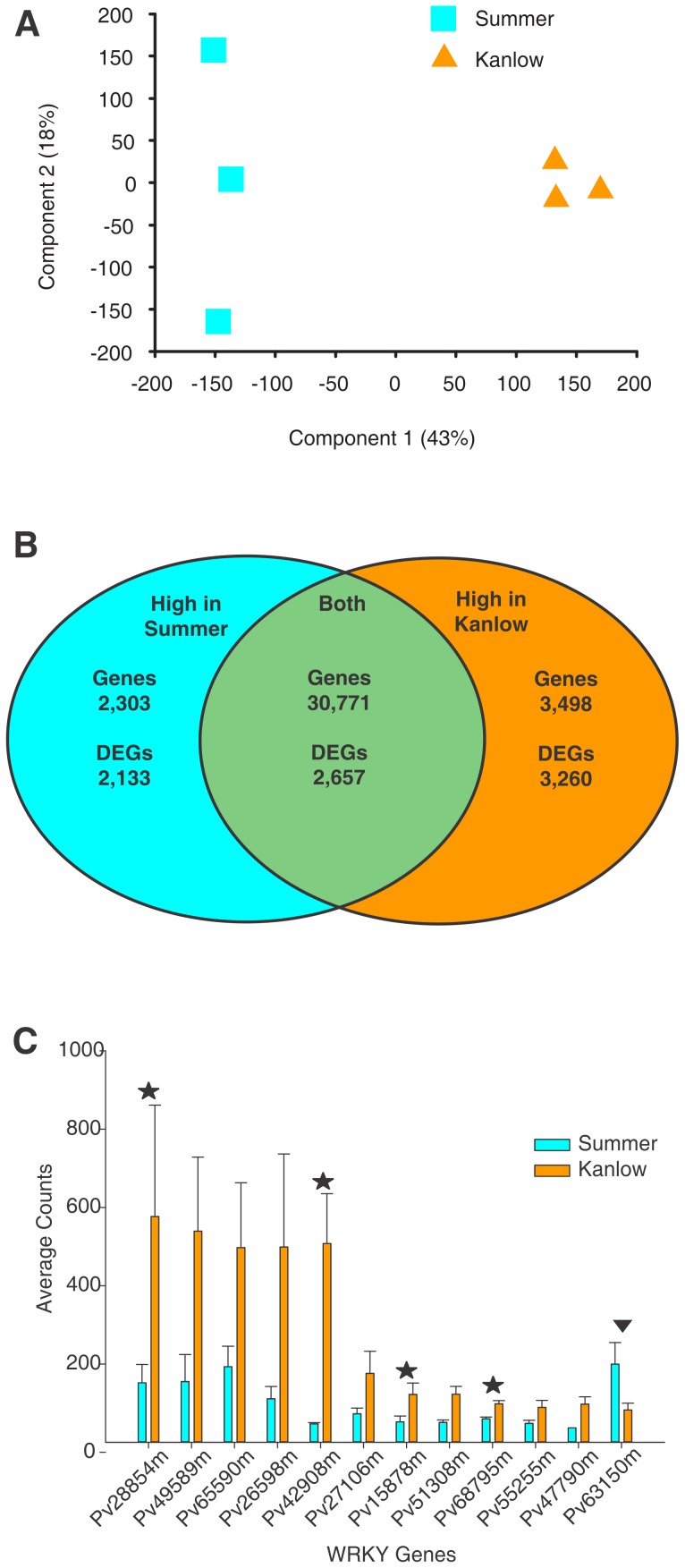
Transcriptomes of Kanlow and Summer plants are different, and show differential enrichment in WRKY genes. (**A**) PCA of transcriptomes of the three biological replicates obtained from each ecotype. Kanlow (orange triangle) were differentiated from Summer (cyan squares) by the first component. (**B**) Venn diagram of numbers of common and highly expressed genes in Summer (log_2_FC<-1) and Kanlow (log_2_FC>1), including DEGs in the Kanlow (orange) and Summer (cyan) datasets. (**C**) Mean counts for twelve transcripts identified as WRKY transcription factors. Stars above orange bars (Kanlow) identify putative switchgrass orthologs of WRKY factors involved in defense response in other systems. The inverted triangle over a cyan bar identifies a WRKY ortholog to an Arabidopsis gene involved in senescence that is upregulated in Summer crowns and rhizomes.

EdgeR analysis identified a total of 8,050 genes that were differentially expressed (File S2) between Kanlow and Summer crown and rhizomes at an FDR<0.05. A principal component analysis (PCA) of this expression dataset effectively separated the three Kanlow samples from the three Summer samples (Figure 1A). Component 1 accounted for 43% of the variance and separated the two cultivars. Component 2 accounted for approximately 18% of the variance and separated the three Summer samples. A Venn diagram of the total number of genes found in the Summer and Kanlow datasets is shown in Figure 1B. In total transcripts were detected for 36,572 genes. Of this total ∼84% (30,771 genes) were common to both populations and 2,303 and 3,498 genes were unique to the Summer and Kanlow datasets respectively (Figure 1B). About 41% of the differentially expressed genes (DEGs) had two-fold or greater average expression in Kanlow, and ∼26% had two-fold or greater expression in Summer (Figure 1B).

A more detailed evaluation of the DEGs was performed to identify changes in transcript abundance for three plant-specific classes of transcription factors, namely MYBs, NACs and WRKYs which are known to have multiple functions in cellular processes [36]–[40]. A scan of the switchgrass genome based on Pfam PF03106 (WRKY), PF00249 (MYB) and PF02365 (NAC) domains [41], identified 178 WRKYs (∼89 WRKYs per diploid genome); 324 MYBs (∼162 MYBs per diploid genome); and 239 NACs (∼120 NACs per diploid genome). Similar searches for WRKY members in other species gave the following results: Arabidopsis – 72; rice – 94 and 82 in *Brachypodium*. Using a similar approach for MYBs and NACs in other species, we found: Arabidopsis – 256/112 (MYBs/NACs); rice – 233/140 (MYBs/NACs), and Brachypodium – 200/100 (MYBs/NACs). Based on the relative numbers of these genes found in model plants, we expect that approximately 80–90% of the orthologous switchgrass genes were identified by this search. A total of 199 MYBs (30 DEG) and 108 NACs (20 DEG) were found in the expression datasets. In all 119 WRKYs (∼67% of the all WRKY genes identified in the genome) were found in the Summer and Kanlow datasets. Of these 119 genes, a total of 12 were differentially expressed at an FDR<0.05 (Figure 1D). A database search indicated that orthologs to four of the WRKY genes upregulated in Kanlow (identified by a star symbol in Figure 1C) were involved in responses to pathogens. In contrast, the ortholog to one WRKY gene upregulated in Summer and has been demonstrated to be an important mediator of cellular responses to senescence (inverted triangle).

### Metabolite analysis differentiates Kanlow from Summer crowns and rhizomes

**Figure 2:**
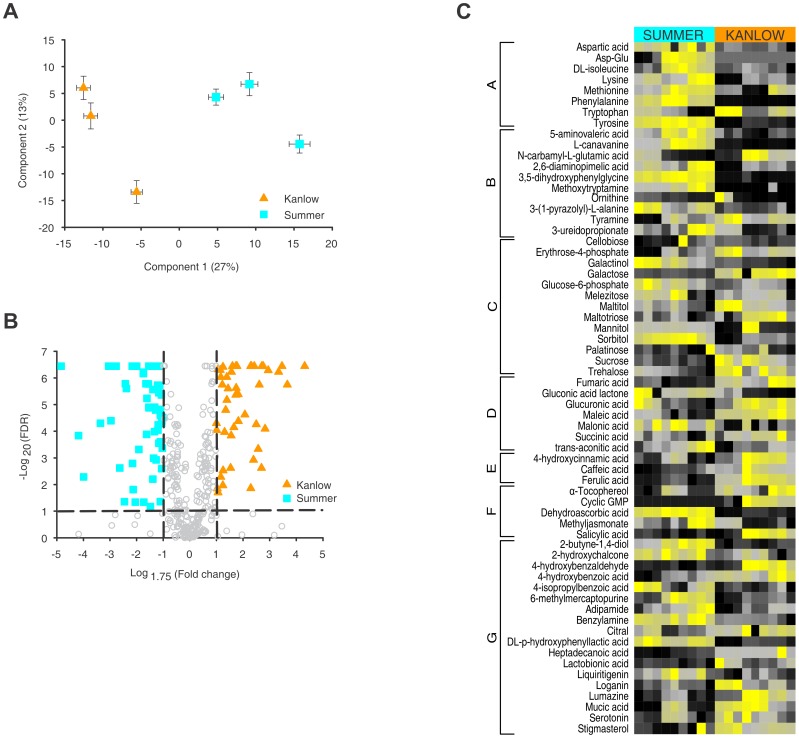
Metabolite profiling reveals ecotype specific differences in crown and rhizome tissues. (**A**) PCA of overall metabolite profiles observed by GCMS for for each of three individual genotypes within each cultivar. Kanlow tissue extracts (orange triangles) are separated from Summer tissue extracts (cyan squares) by the first component. Two-way error bars are shown for each plant that was based on nine separate GCMS runs for each sample. (**B**) Volcano plot showing the log_10_FDR versus the log_1.75_ fold change in peak area for major ions for all metabolites detected by GCMS. Significant differences were observed for several metabolites between the two cultivars (Kanlow orange triangles) and Summer (cyan squares). Grey circles are metabolites that failed to show sufficient differences in ion area of had an FDR value of >0.05. (**C**) Heat map shows marked differences in tissue abundances of selected metabolites in Kanlow and Summer crowns and rhizomes. Data are the average of triplicate injections from each of three separate extractions from the three biological replicates of each cultivar. Scale is from high abundance (yellow) to low abundance (black) for each metabolite. Data were subjected to one-way hierarchical clustering based on cultivar, and metabolites were manually reordered into more biologically related groupings (A–G).

Principal component analysis of a broad-based polar metabolite profiling of Kanlow and Summer crown and rhizome extracts using GC-MS revealed that Summer and Kanlow extracts were clearly differentiated by the first principal component, which explained 27% of the variance (Figure 2A). The second component differentiated among the individual genotypes within each cultivar and explained an additional 13% of the variance. Concentration ratios, approximated by major ion count, were coupled with differences that had an FDR<0.05 to generate a volcano plot of the metabolite data (Figure 2B). Concentration ratios between the cultivars were considered meaningful if the averaged ion counts for a metabolite had a ratio greater than 1.75 or less than 1.75^−1^. The stricter criteria resulted in 66 metabolites with sufficient FDR values and ion area ratios in Summer and 47 metabolites in Kanlow. Hierarchical two-way clustering was used to generate an initial color map of the overall metabolite profile (Figure S2 in File S1). Following this analysis, we generated the heat map shown in Figure 2C by using one-way hierarchical clustering based on cultivar, and manually reordered metabolites into more biologically related groupings which could be indicative of differences in their metabolism (Figure 2C A–G). In Summer plants, levels of several amino acids (Figure 2C group A), amine derivatives (group B) and some sugar alcohols (group C) were significantly higher. In contrast, erythrose-4-phosphate, maltotriose, sucrose and trehalose were detected in greater levels in Kanlow crowns and rhizomes. Further significant differences (based on an FDR<0.05) in the metabolism between Kanlow and Summer rhizomes were evident in the observed levels of several organic acids such as glucuronic acid, a precursor of xylans [42], and a range of secondary metabolites, including phenolic acids (Figure 2C, group D,E). Summer tissues also appeared to be in a more oxidized state as compared to Kanlow crowns and rhizomes as they contained significantly higher levels of dehydroascorbic acid, gluconic acid lactone, and lower concentrations of α-tocopherol, (Figure 2C, group D, F).

### Nexus between gene-set enrichment and metabolite analyses

As a prelude to gene-set enrichment analysis (GSEA), 124 gene sets (Table S3 in File S1) were generated for the predicted transcriptome available in the PviDraft0 using Blast2GO and the Kyoto Encyclopedia of Genes and Genomes (KEGG; http://www.genome.jp/kegg/). These gene sets were then used through GSEA to identify specific pathways differentially regulated in the crown and rhizome tissues of Kanlow and Summer plants. A total of 24 metabolic pathways (FDR<0.2) were different between the two ecotypes (Table S4 in File S1). In Kanlow tissues, there was an apparent upregulation of phenylpropanoid biosynthesis (cell wall accretion), starch, sucrose and fatty acid metabolism. Within Summer plants, there was enrichment (FDR<0.001) in transcripts for enzymes involved in diterpenoid biosynthesis, and a somewhat weaker upregulation for transcripts for enzymes associated with the biosynthesis of unsaturated fatty acids (FDR<0.193). A number of metabolic pathways associated with the degradation and scavenging of carbon compounds were also enriched in Summer crowns and rhizomes (Table S4 in File S1), providing some consistency with metabolite data.

**Figure 3:**
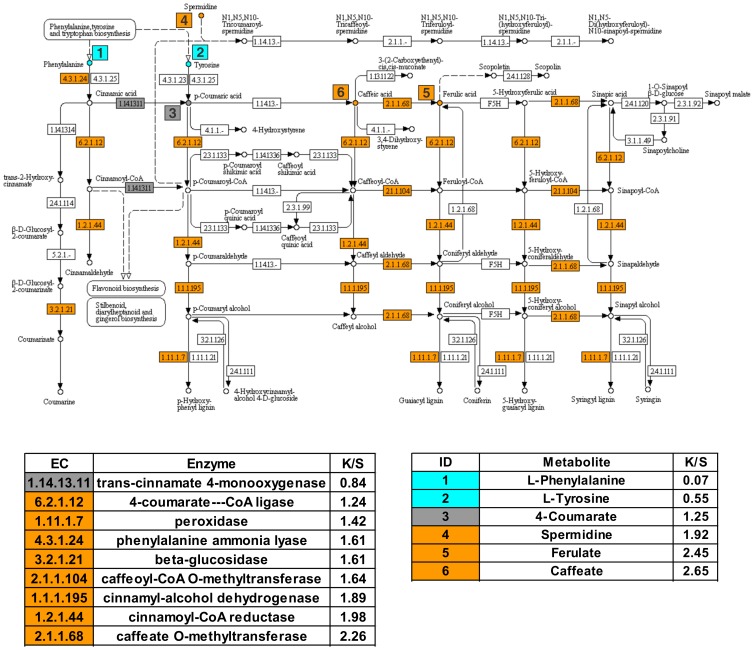
Transcripts for enzymes and several metabolites associated with the phenylpropanoid pathway are significantly enhanced in Kanlow crowns and rhizomes. Orange boxes identify individual enzymes upregulated in Kanlow. Boxes with EC numbers and relative enrichment of transcripts (Kanlow/Summer, K/S) are shown. Boxes with numbers indicate metabolites associated with this pathway identified by GCMS. Cyan (higher levels in Summer tissues), orange (higher in Kanlow tissues) and grey boxes (not significantly different).

In Kanlow tissues, there was significant enrichment in the transcripts coding for enzymes in the phenylpropanoid (lignin biosynthesis) pathway as compared to Summer tissues (Figure 3). Key metabolites caffeic acid and ferulic acid were enriched in Kanlow tissues. In Summer crowns and rhizomes, levels of Phe and Tyr, precursors for the phenylpropanoid pathway were significantly greater, consistent with the transcriptomic data.

### Enzyme activity, protein levels and enrichment of key transcription factors confirm GSEA of phenylpropanoid pathway

**Figure 4:**
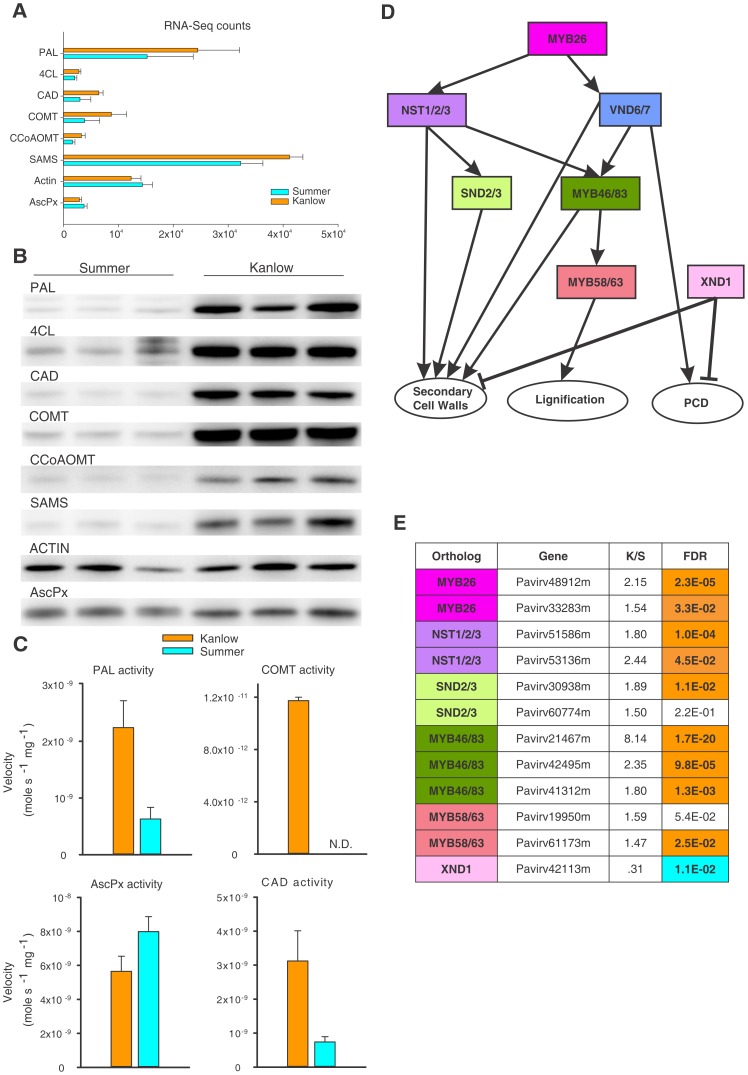
Transcript, protein and enzyme activities confirm downregulation of phenylpropanoid pathway and upregulation of ascorbate peroxidase in Summer crowns and rhizomes. (**A**) Transcript abundances for all transcripts identified as belonging to a specific gene by Blast2GO. PAL, phenylalanine ammonia lyase; 4CL, 4-coumarate-CoA ligase; CAD, cinnamyl alcohol dehydrogenase; COMT, caffeoyl-O-methyltransferase; CCoAOMT, caffeoyl-CoA 3O-methyltransferase; SAMS, s-adenosylmethionine synthetase; AscPX, ascorbate peroxidase. Orange bars = Kanlow; Cyan bars = Summer. (**B**) Immunblots of crown and rhizome extracts separated by SDS-PAGE. Extracts from three biological replicates for each cultivar are shown. These were the same tissues used for transcriptome and metabolite analyses. Other designations as described for panel A. (**C**) Enzyme activities of PAL, COMT, AscPx and CAD. Orange bars = Kanlow; Cyan bars = Summer. Other designations as described for panel **A**. (**D**) A map of potential relationships of key transcription factors that are known to play a major role in plant cell wall developmental processes. Each specific factor is shown in a different colored box. Figure is based on work described by Zhao and Dixon (2011). (**E**) Best switchgrass orthologs to transcription factors shown in (D). Boxes are colored identically to Panel D above. The switchgrass loci are identified and the FDR<0.05 values are shown in the last column. For the last column, boxes in orange color (transcripts significantly upregulated in Kanlow), cyan color (significantly upregulated in Summer), uncolored, not significant.

The phenylpropanoid pathway was targeted to understand if the changes documented for transcript abundance (Figure 3) were also evident at the protein level. Transcripts for several genes associated with the phenylpropanoid pathway were significantly enhanced in Kanlow crowns and rhizomes as compared to the Summer plants. These included PAL, 4-coumarate-CoA ligase (4-CL), CAD, COMT and caffeoyl CoA 3-O-methyltransferase (CCoAOMT) (Figure 4A). It should be noted that transcript abundance for a called gene in Figure 4A is representative of all sequences assigned to this class of gene by Blast2GO, and is likely to contain transcripts for one or more related genes within a given gene family. Abundances varied over an order of magnitude, suggesting that differential regulatory mechanisms might exist for these individual genes. Transcript levels for actin were not significantly different (Figure 4A). AscPx transcripts were slightly elevated in Summer tissues as compared to Kanlow. AscPx is involved in detoxification of ROS in cells. Next, antibodies generated to select enzymes in the lignin biosynthesis pathway were utilized and showed that apparent protein levels for PAL, 4-CL, CAD, COMT and CCoAOMT were lower in all three biological replicates of Summer crown and rhizome extracts as compared to Kanlow extracts (Figure 4B). Levels for s-adenosyl methionine synthetase (SAMS), an enzyme that produces S-adenosyl methionine, which is a substrate for COMT and CCoAOMT, was also depressed in Summer crowns and rhizomes. Signal intensity for actin (used as a loading control) was similar for the Kanlow extracts and two of the Summer extracts, and diminished in one Summer extract. However, all the other proteins probed were essentially identical to the levels detected in the two other Summer plants (Figure 4B). The consistent differences observed in the transcript abundances and protein levels were also mirrored in the activities of PAL, CAD and COMT which were significantly lower or not detected in extracts from Summer tissues as compared to extracts prepared from the Kanlow crowns and rhizomes. AscPx activities were greater in Summer plants as compared to the Kanlow plants (Figure 4C) in agreement with the transcript and protein data. These data suggested that transcription factors known to affect the phenylpropanoid pathway [43] could be similarly regulated in these plants at this harvest date. The expected relationships of the key transcription factors involved in secondary cell wall deposition and lignification, adapted from [43], is shown in Figure 4D. Individual regulatory genes are color coded in boxes and the cellular pathways they impact are shown in ovals. Arrows connect the putative relationships between these gene products in model systems and to their respective cellular processes (ovals, Figure 4D). Although MYB 26 has been depicted as a master regulator in Figure 4D, its overall role is not as well established as for the other regulatory genes [43]. The XND1 gene (pink box) is a negative regulator of xylogenesis, and inhibits programmed cell death (PCD) and related processes in Arabidopsis [44]. The best switchgrass orthologs to these individual transcription factors (color coded identically in Figure 4D and 4E) which positively regulate secondary cell growth and/or lignification exhibited greater ratios of transcript abundance (Kanlow/Summer; K/S). Most of these genes were significantly upregulated (FDR<0.05) in Kanlow crowns and rhizomes (highlighted in orange). In contrast, transcripts for the XND1 ortholog were significantly greater in Summer tissues (Figure 4E).

## Discussion

In this study the dynamic aspects of the crown and rhizome transcriptome and metabolome in switchgrass has been explored for the first time. Analysis at this single harvest date revealed interesting insights into the metabolism of the crowns and rhizomes obtained from lowland Kanlow and upland Summer plants which differ in their photoperiod maturity response at the field site used for this experiment [3]. The work reported here complements several recent articles have expanded the available molecular resources for switchgrass and have begun the process of understanding switchgrass cellular processed within the context of its genomic complexity [16], [45]–[50].

The use of NGS coupled to bioinformatic analyses allowed approximately 80% of all reads to be effectively mapped to the switchgrass genome. Subsequent bioinformatic analyses indicated that these transcripts arose from 36,572 genes after filtering, which is ∼56% of the total loci identified so far in the switchgrass genome, suggesting that the mapping and identification processes were relatively robust. It is likely that potentially interesting features could have been missed due to the mapping of reads to the unfinished contig-based switchgrass genome assembly. Thus, mapping of transcripts to incomplete genes, gene sequences with no discernible domains or near the end of contigs would have been excluded from our dataset. However, these data provided adequate depth to begin an understanding of the metabolism of the crowns and rhizomes from the two contrasting switchgrass populations used in this study.

RNA-Seq of the transcriptomes provided a first broad-scale means to directly assess the metabolic status of these plants. PCA showed that the transcriptomes were clearly differentiated by the first component, indicating that between population (cultivar) differences were greater than within population differences. Both switchgrass cultivars are a heterogenous collection of genotypes [11] and the data for Summer plants would support this fact. The greater variation observed between the Summer plants could be indicative of differences in the timing of aerial senescence in these genotypes and its effect on crown and rhizome metabolism. In contrast, the transcriptomes of the Kanlow plants appeared to be more tightly clustered. Indeed the metabolomes also reflected this analysis. The two types of relatively non-biased large-scale approaches (RNA-Seq and GCMS) yielded similar conclusions with regard to the divergent metabolism in the Summer plants as compared to the Kanlow plants at this specific harvest date. Environmental conditions prior to the harvest date indicated that the field site had received adequate rainfall and had not experienced freezing temperatures minimizing abiotic stresses on these plants. Although these data are supportive of the notion that the differences in metabolism observed in the crowns and rhizomes were more reflective of developmental events, it would be difficult to rule out biotic and abiotic stresses these plants may have experienced during growth and during sample handling.

Differential enrichment of transcripts, metabolites and gene-sets were observed between the two populations. Analysis of transcription factors such as WRKYs [51]–[53] which impact an extensive array of plant cellular processes was undertaken. Approximately ∼67% of the all WRKYs tentatively identified in the switchgrass genome were represented in the total transcriptomic datasets, indicative of their potential role in switchgrass crowns and rhizomes. Within this larger group, of the nine *WRKY* genes significantly upregulated in Kanlow plants, four genes appeared to be orthologs to Arabidopsis WRKYs with a role in the defense response to pathogens [54]–[56]. In contrast, the WRKY factor enriched in Summer tissues have been implicated as a negative regulator of leaf senescence [55]. Although the actual roles of individual switchgrass WRKYs remain to be explored, they could be reflective of other changes observed at the metabolite and GSEA levels. Summer crowns and rhizomes were enriched in some oxidized compounds and appeared to contained lower levels of α-tocopherol [57], and phenolic acids that could potentially function as antioxidants [58], [59]. Although these data show a link between the transcriptomic and metabolomic datasets, much work will be needed to unequivocally solve these interrelationships.

Transcriptomic and metabolomic data were consistent with the hypothesis that Kanlow tissues were actively utilizing carbon in contrast to Summer crowns and rhizomes. Sucrose and trehalose levels as well the GSEA results showing the enrichment of sucrose-starch metabolism pathways in Kanlow crowns were consistent with this hypothesis. Also other downstream biosynthetic pathways that rely on carbon skeletons derived from sugars were also enriched in Kanlow tissues. Most notably, there was a strong enrichment in pathways linked to cell wall accretion and growth occurring in Kanlow plants relative to the Summer plants. Data shown in Figure 4 effectively linked transcript and metabolite datasets to the underlying protein activities, at least for a portion of the lignin biosynthesis pathway which showed higher activity in Kanlow tissues. Certain phenolic metabolites, namely cinnamic acid, ferulic acid and caffeic acid that are associated with monolignol synthesis [31], [60], were also more enriched in Kanlow consistent with enhanced flow of carbon through this pathway. In contrast, the amino acid precursors for this pathway, Phe and Tyr, were significantly enriched in Summer crowns and rhizomes which suggested that down-regulation of the phenylpropanoid pathway enzymes could have led to the increased accumulation of these amino acids. The significant up-regulation in transcript abundance for the switchgrass genes identified as potential orthologs of master transcription factors that regulate secondary cell wall biogenesis and lignification, based on [43], in Kanlow plants was striking and validated the other protein and metabolite findings. In contrast, transcripts for the putative switchgrass NAC gene *XND1* were significantly greater in Summer tissues, suggestive of reduction in xylogenesis, and possibly in growth of tiller initials and rhizomes. In Arabidopsis, XND1 acts as a repressor of xylogenesis and programmed cell death (PCD) [44].

**Figure 5:**
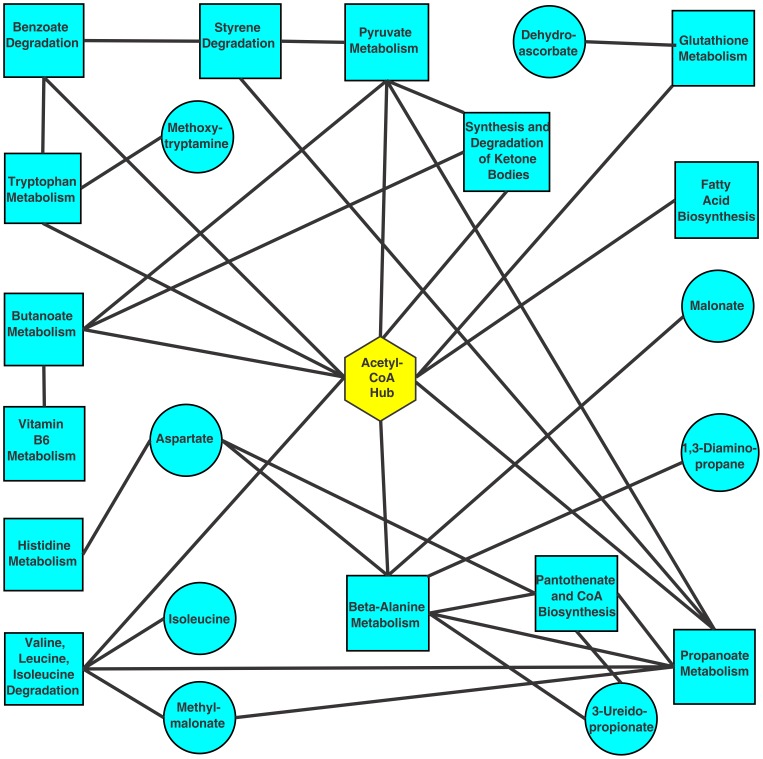
Acetyl-CoA appears to be a central hub connecting diverse pathways upregulated in Summer crowns and rhizomes. Cyan squares are KEGG pathways found to be upregulated in Summer plants by GSEA, and cyan circles are, and metabolites that were elevated in Summer plants relative to Kanlow plants. Edges connecting pathways (squares) to metabolites (circle) indicate that the given metabolite is found in the connected pathway. Pathways with direct connections in KEGG are also connected by edges. Acetyl-CoA (diamond) could be a potential linker molecule among these diverse pathways and is suggested to be a possible metabolic hub in Summer crowns and rhizomes entering dormancy.

A number of KEGG pathways were found in Summer crowns and rhizomes that were enriched for transcripts, although not all enzymes were populated, unlike the more clear-cut observations on the phenylpropanoid pathway in Kanlow tissues. In trying to establish the possible connections between these diverse metabolic pathways, it became apparent that most possessed a potential common metabolite in acetyl-CoA (Figure 5). Potentially, scavenging of carbon compounds to form acetyl-CoA could represent a means to meet energy demands needed for maintaining cellular health in the possible absence of photosynthate delivery from the senescent shoots in Summer plants, which are consistent with results obtained for leafy spurge [61]. Acetyl-CoA serves as a key link between cellular metabolism, energetics and the acetylation of proteins in animal cells [62]. Similar mechanisms appear to be operating in plants [63]–[65], suggesting that a deeper understanding of the flux of carbon through a acetyl-CoA hub could provide new insights into the metabolism of crowns and rhizomes accompanying the seasonal growth habit of switchgrass and related perennial plants. Unfortunately, the metabolite analysis protocol used in this study cannot detect acetyl-CoA. Future use of other platforms such as LC-ms/ms might yield information on these more unstable metabolites.

## Conclusions

Data presented in this manuscript showed that differences existed in the metabolite and transcript profiles in the crowns and rhizomes obtained from field-grown switchgrass plants belonging to two populations with divergent photoperiod responses. Crowns and rhizomes obtained from the more southern adapted cultivar Kanlow appeared to be in a “growth-mode” with enrichment in gene-sets associated with biosynthesis and accretion of tissues. In contrast, the northern adapted cultivar Summer appeared to have entered a more quiescent state with greater enrichment of transcripts and metabolites favoring channeling of carbon skeletons through an acetyl-CoA hub. Our findings provide an initial framework to understand differences in crown and rhizome metabolism which could be exploited to enhance the latitudinal adaptation of diverse switchgrass populations.

## Supporting Information

File S1 Table S1 - Mapping summary for switchgrass crown and rhizome samples analyzed by HTS; Table S2 - Annotation of all transcripts using Blast2GO; Table S3 - List of 124 KEGG Pathways populated by Blast2GO analysis of the switchgrass draft genome; Table S4 - Gene Set Enrichment Analysis Results. KEGG pathways with an FDR<0.2 are shown. Transcript observed for individual enzymes in a given pathway are tabulated (enzymes); Figure S1 - Temperature and rainfall at the field site prior to and post-harvest; Figure S2 - Heat map of all major ions detected by GCMS using two-way hierarchical clustering. Yellow = high, Black = low. Data are for three independent extractions from three plants.
(PDF)

File S2 **Excel file with list of differentially expressed genes (DEGs).**
(XLSX)
