# Supplementary material for: Contrasting Metabolism in Perenniating Structures of Upland and Lowland Switchgrass Plants Late in the Growing Season
Source: PLoS One. 2014 Aug 18;9(8):e105138. doi: 10.1371/journal.pone.0105138 (PMC4136849; doi:10.1371/journal.pone.0105138)

Table S1. Mapping summary for switchgrass crown and rhizome samples analyzed by HTS

Plant ID	Summer1	Summer2	Summer3	Kanlow1	Kanlow2	Kanlow3
Total Reads	26,158,420	26,000,859	26,818,136	24,539,570	27,127,805	27,090,340
Total Mapped to Genome	20,032,371	20,879,066	20,548,960	20,463,917	22,290,859	21,847,400
Percent of Total	76.6	80.3	76.6	83.4	82.2	80.6
Multi-Mapped Reads	3,179,885	4,163,674	3,198,823	2,624,806	3,254,418	3,051,917
Percent of Total	12.2	16.0	11.9	10.7	12.0	11.3
Uniquely Mapped Reads						
In Annotated Genes	14,573,318	14,674,977	14,936,660	15,198,819	16,141,080	16,024,441
Percent of Total	55.7	56.4	55.7	61.9	59.5	59.2
In Unannotated Regions	2,279,168	2,040,415	2,416,477	2,640,292	2,895,361	2,771,042
Percent of Total	8.7	7.8	9.0	10.8	10.7	10.2

Table S2. Annotation of all transcripts using Blast2GO

Total Transcripts	No Blastx Hit*	no GO Terms	not Annotated**	Annotated
65,878	4,986	6,594	8,811	45,487

Total EC Assigned	Transcripts with EC	Molecular Function	Biological Processes
15,507	13,406	35,438	28,115

KEGG Pathways

Enzymes in KEGG Paths	KEGG Paths Populated
1,347	138

* at an e-value threshold of 1×10^{-7} or lower; ** at an e-value threshold of 1×10^{-15} or lower

Table S3. List of 124 KEGG Pathways populated by Blast2GO analysis of the switchgrass draft genome used for Gene Set Enrichment Analysis

NAME	# Enzymes
PURINE METABOLISM	52
AMINO SUGAR AND NUCLEOTIDE SUGAR METABOLISM	40
ARGININE AND PROLINE METABOLISM	40
STARCH AND SUCROSE METABOLISM	38
PYRIMIDINE METABOLISM	32
CYSTEINE AND METHIONINE METABOLISM	30
GLYCOLYSIS / GLUCONEOGENESIS	26
ALANINE, ASPARTATE AND GLUTAMATE METABOLISM	26
GLYCEROPHOSPHOLIPID METABOLISM	25
GLYCINE, SERINE AND THREONINE METABOLISM	25
PORPHYRIN AND CHLOROPHYLL METABOLISM	25
METHANE METABOLISM	24
PYRUVATE METABOLISM	23
AMINOACYL-TRNA BIOSYNTHESIS	22
CARBON FIXATION IN PHOTOSYNTHETIC ORGANISMS	21
NITROGEN METABOLISM	21
INOSITOL PHOSPHATE METABOLISM	21
VALINE, LEUCINE AND ISOLEUCINE DEGRADATION	20
FRUCTOSE AND MANNOSE METABOLISM	20
GLYOXYLATE AND DICARBOXYLATE	20

METABOLISM	
TERPENOID BACKBONE BIOSYNTHESIS	20
PHENYLALANINE, TYROSINE AND TRYPTOPHAN BIOSYNTHESIS	19
GLUTATHIONE METABOLISM	18
GLYCEROLIPID METABOLISM	18
TYROSINE METABOLISM	18
GALACTOSE METABOLISM	17
PHOSPHATIDYLINOSITOL SIGNALING SYSTEM	17
BETA-ALANINE METABOLISM	16
CITRATE CYCLE (TCA CYCLE)	16
PROPANOATE METABOLISM	16
CARBON FIXATION PATHWAYS IN PROKARYOTES	16
PHENYLALANINE METABOLISM	15
PENTOSE PHOSPHATE PATHWAY	15
DRUG METABOLISM - OTHER ENZYMES	15
BUTANOATE METABOLISM	15
PANTOTHENATE AND COA BIOSYNTHESIS	15
PENTOSE AND GLUCURONATE INTERCONVERSIONS	14
FATTY ACID METABOLISM	14
DITERPENOID BIOSYNTHESIS	14
SULFUR METABOLISM	13
ONE CARBON POOL BY FOLATE	13
ASCORBATE AND ALDARATE METABOLISM	12
SPHINGOLIPID METABOLISM	12
N-GLYCAN BIOSYNTHESIS	12
LYSINE DEGRADATION	11
TRYPTOPHAN METABOLISM	11
CYANOAMINO ACID METABOLISM	11
UBIQUINONE AND OTHER TERPENOID-QUINONE	11

BIOSYNTHESIS	
HISTIDINE METABOLISM	11
ALPHA-LINOLENIC ACID METABOLISM	10
SELENOCOMPOUND METABOLISM	10
NICOTINATE AND NICOTINAMIDE METABOLISM	10
FLAVONOID BIOSYNTHESIS	10
STEROID BIOSYNTHESIS	10
AMINOBENZOATE DEGRADATION	9
RIBOFLAVIN METABOLISM	9
STEROID HORMONE BIOSYNTHESIS	9
FATTY ACID BIOSYNTHESIS	9
VALINE, LEUCINE AND ISOLEUCINE BIOSYNTHESIS	9
VARIOUS TYPES OF N-GLYCAN BIOSYNTHESIS	9
LYSINE BIOSYNTHESIS	9
STREPTOMYCIN BIOSYNTHESIS	9
PHENYLPROPANOID BIOSYNTHESIS	8
ARACHIDONIC ACID METABOLISM	8
BIOSYNTHESIS OF UNSATURATED FATTY ACIDS	8
FOLATE BIOSYNTHESIS	8
DRUG METABOLISM - CYTOCHROME P450	7
THIAMINE METABOLISM	7
METABOLISM OF XENOBIOTICS BY CYTOCHROME P450	7
OXIDATIVE PHOSPHORYLATION	7
OTHER GLYCAN DEGRADATION	7
RETINOL METABOLISM	7
TROPANE, PIPERIDINE AND PYRIDINE ALKALOID BIOSYNTHESIS	7
VITAMIN B6 METABOLISM	7
BENZOATE DEGRADATION	6
FATTY ACID ELONGATION	6

CAROTENOID BIOSYNTHESIS	6
ETHER LIPID METABOLISM	5
GLYCOSAMINOGLYCAN BIOSYNTHESIS - HEPARAN SULFATE	5
STYRENE DEGRADATION	5
LIPOPOLYSACCHARIDE BIOSYNTHESIS	5
LINOLEIC ACID METABOLISM	4
GLYCOSPHINGOLIPID BIOSYNTHESIS - GLOBO SERIES	4
ISOQUINOLINE ALKALOID BIOSYNTHESIS	4
TAURINE AND HYPOTAURINE METABOLISM	4
GERANIOL DEGRADATION	4
SYNTHESIS AND DEGRADATION OF KETONE BODIES	4
BIOTIN METABOLISM	4
GLYCOSAMINOGLYCAN BIOSYNTHESIS - CHONDROITIN SULFATE	3
GLYCOSAMINOGLYCAN DEGRADATION	3
INDOLE ALKALOID BIOSYNTHESIS	3
LIMONENE AND PINENE DEGRADATION	3
GLYCOSPHINGOLIPID BIOSYNTHESIS - GANGLIO SERIES	3
CHLOROALKANE AND CHLOROALKENE DEGRADATION	3
CAFFEINE METABOLISM	3
ZEATIN BIOSYNTHESIS	3
NOVOBIOCIN BIOSYNTHESIS	3
POLYKETIDE SUGAR UNIT BIOSYNTHESIS	3
CHLOROCYCLOHEXANE AND CHLOROBENZENE DEGRADATION	3
TOLUENE DEGRADATION	3
PHOSPHONATE AND PHOSPHINATE METABOLISM	3

OTHER TYPES OF O-GLYCAN BIOSYNTHESIS	3
PEPTIDOGLYCAN BIOSYNTHESIS	3
T CELL RECEPTOR SIGNALING PATHWAY	2
GLYCOSPHINGOLIPID BIOSYNTHESIS - LACTO AND NEOLACTO SERIES	2
GLUCOSINOLATE BIOSYNTHESIS	2
CAPROLACTAM DEGRADATION	2
C5-BRANCHED DIBASIC ACID METABOLISM	2
STILBENOID, DIARYLHEPTANOID AND GINGEROL BIOSYNTHESIS	2
FLAVONE AND FLAVONOL BIOSYNTHESIS	2
LIPOIC ACID METABOLISM	2
BUTIROSIN AND NEOMYCIN BIOSYNTHESIS	2
FLUOROBENZOATE DEGRADATION	2
D-GLUTAMINE AND D-GLUTAMATE METABOLISM	2
ATRAZINE DEGRADATION	2
PRIMARY BILE ACID BIOSYNTHESIS	2
MTOR SIGNALING PATHWAY	1
NAPHTHALENE DEGRADATION	1
BIOSYNTHESIS OF VANCOMYCIN GROUP ANTIBIOTICS	1
ETHYLBENZENE DEGRADATION	1
PHOTOSYNTHESIS	1
GLYCOSYLPHOSPHATIDYLINOSITOL(GPI)-ANCHOR BIOSYNTHESIS	1
BIOSYNTHESIS OF TERPENOIDS AND STEROIDS	1
BETALAIN BIOSYNTHESIS	1

Table S4: Gene Set Enrichment Analysis Results. KEGG pathways with an FDR <0.2 are shown. Transcript observed for individual enzymes in a given pathway are tabulated (enzymes).

Up-regulated in Summer

Pathway	FDR	Enzymes
DITERPENOID BIOSYNTHESIS	0.000	14
VALINE, LEUCINE AND ISOLEUCINE DEGRADATION	0.001	20
DRUG METABOLISM - CYTOCHROME P450	0.012	7
METABOLISM OF XENOBIOTICS BY CYTOCHROME P450	0.012	7
FATTY ACID ELONGATION	0.013	6
BUTANOATE METABOLISM	0.014	15
GERANIOL DEGRADATION	0.020	4
VITAMIN B6 METABOLISM	0.024	7
BENZOATE DEGRADATION	0.034	6
STYRENE DEGRADATION	0.039	5
GLUTATHIONE METABOLISM	0.051	18
HISTIDINE METABOLISM	0.058	11
VALINE, LEUCINE AND ISOLEUCINE BIOSYNTHESIS	0.063	9
SYNTHESIS AND DEGRADATION OF KETONE BODIES	0.066	4
BETA-ALANINE METABOLISM	0.067	16
PROPANOATE METABOLISM	0.073	16
PYRUVATE METABOLISM	0.079	23
TRYPTOPHAN METABOLISM	0.080	11
PANTOTHENATE AND COA BIOSYNTHESIS	0.188	15
BIOSYNTHESIS OF UNSATURATED FATTY ACIDS	0.193	8

Up –regulated in Kanlow

Pathway	FDR	Enzymes
PHENYLPROPANOID BIOSYNTHESIS	0.001	8

LINOLEIC ACID METABOLISM	0.007	4
STARCH AND SUCROSE METABOLISM	0.007	38
PHENYLALANINE METABOLISM	0.017	15
OTHER GLYCAN DEGRADATION	0.020	7
METHANE METABOLISM	0.033	24
PENTOSE AND GLUCURONATE INTERCONVERSIONS	0.040	14
CAROTENOID BIOSYNTHESIS	0.047	6
GLYCOSAMINOGLYCAN BIOSYNTHESIS - HEPARAN SULFATE	0.050	5
FLAVONOID BIOSYNTHESIS	0.079	10
STREPTOMYCIN BIOSYNTHESIS	0.084	9
SPHINGOLIPID METABOLISM	0.089	12
INOSITOL PHOSPHATE METABOLISM	0.197	21

Figure S1. Temperature and rainfall at the field site prior to and post harvest.

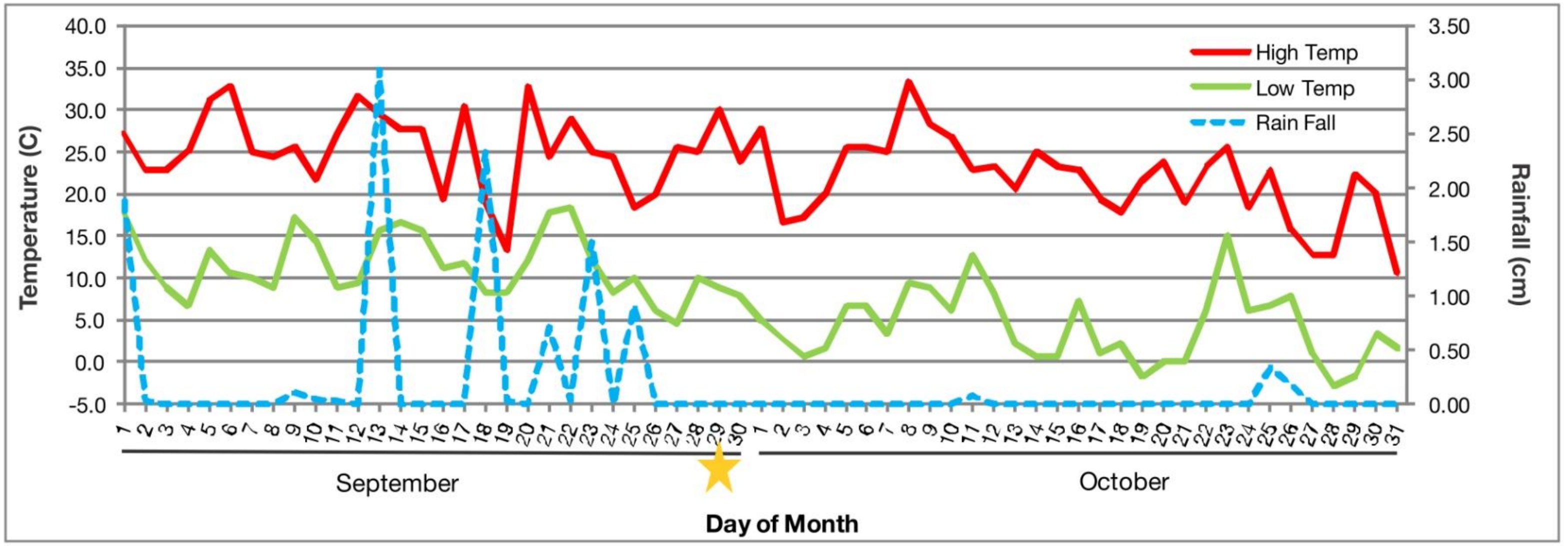

Figure S2. Two way heat map of all features observed by GCMS. Metabolites identified with higher degree of confidence are shown in Fig. 6 Red bar = Kanlow samples; Green bar = Summer samples. Yellow = high, black = low. Data from three independent extractions from each plant sample.

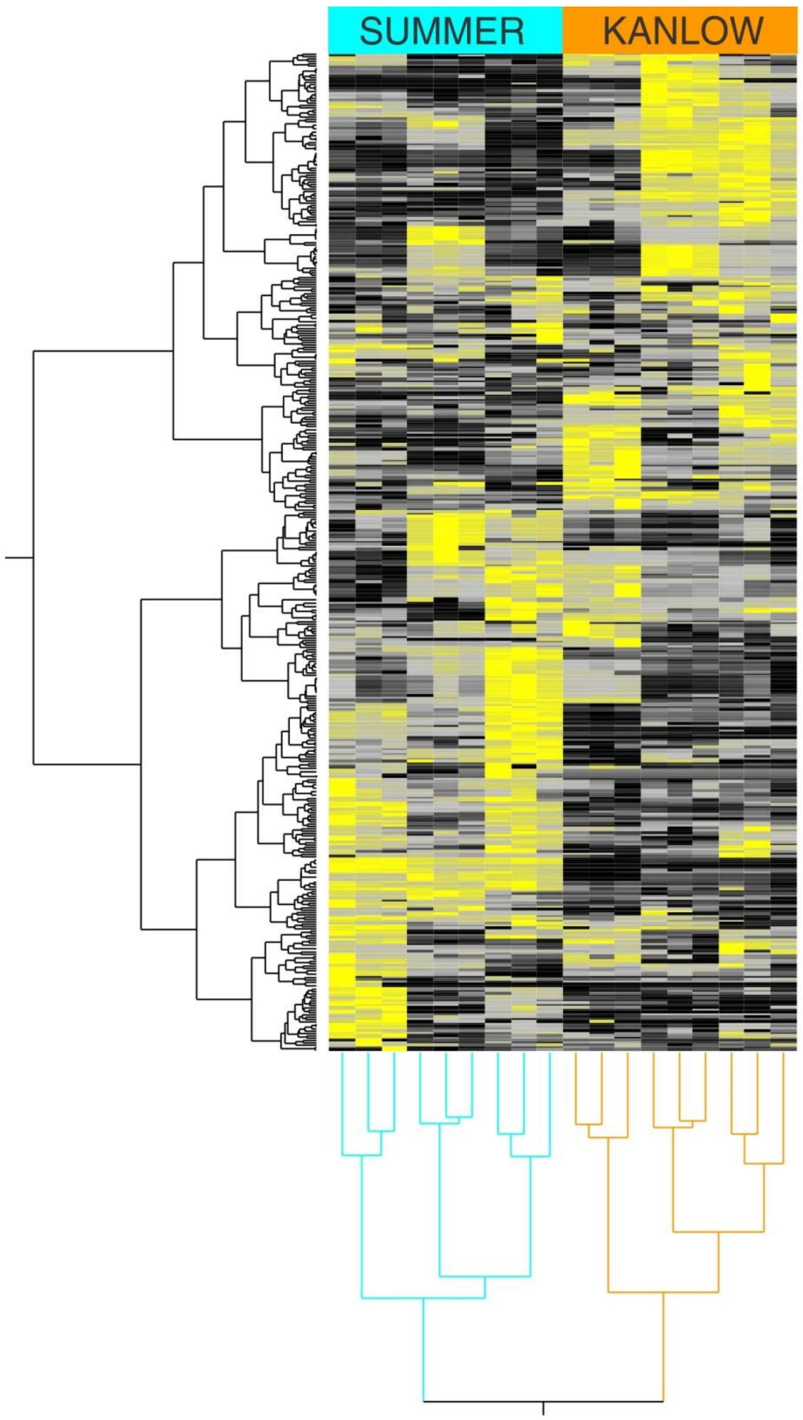

Supplement: File S1 — Table S1 - Mapping summary for switchgrass crown and rhizome samples analyzed by HTS; Table S2 - Annotation of all transcripts using Blast2GO; Table S3 - List of 124 KEGG Pathways populated by Blast2GO analysis of the switchgrass draft genome; Table S4 - Gene Set Enrichment Analysis Results. KEGG pathways with an FDR<0.2 are shown. Transcript observed for individual enzymes in a given pathway are tabulated (enzymes); Figure S1 - Temperature and rainfall at the field site prior to and post-harvest; Figure S2 - Heat map of all major ions detected by GCMS using two-way hierarchical clustering. Yellow = high, Black = low. Data are for three independent extractions from three plants. (PDF) [file pone.0105138.s001.pdf]
